# 2003-2019: explosive spread of enterobacteria producing extended-spectrum beta-lactamases in Bangui Central African Republic

**DOI:** 10.11604/pamj.2021.39.22.28812

**Published:** 2021-05-07

**Authors:** Hugues Sanke-Waïgana, Jean-Robert Mbecko, Gilles Ngaya, Alexandre Manirakiza, Berlioz-Arthaud Alain

**Affiliations:** 1 Clinical Laboratory, Pasteur Institute of Bangui, Bangui, Central African Republic,; 2 Laboratory of Medical and Experimental Bacteriology Pasteur Institute of Bangui, Bangui, Central African Republic,; 3 Epidemiology Unit, Pasteur Institute of Bangui, Bangui, Central African Republic

**Keywords:** Antibiotic resistance, enterobacteria, extended-spectrum beta-lactamases (ESBL), CTX-M, Bangui

## Abstract

**Introduction:**

the spread of enterobacteria producing extended-broad-spectrum beta-lactamases (ESBL) is a global public health-problem. In a study carried in 2003-2005 at the Pasteur Institute in Bangui, 450 enterobacteria were identified in clinical isolates, of which 17 were ESBL (prevalence: 3.78%). The aim of this study was to update this data.

**Methods:**

from May 2018 to April 2019, a total of 941 enterobacteria were isolated and identified under identical conditions of recruitment and with the same techniques used in the previous study: phenotypic identification using Api 20E strips (bioMérieux SA, Marcy-l'Etoile, France) and antimicrobial drug susceptibility using the disk diffusion method (Bio-Rad antibiotic discs, Marnes la Coquette, France). Resistance genes were identified by polymerase chain reaction (PCR) and sequencing.

**Results:**

from May 2018 to April 2019, a total of 941 enterobacteria were isolated of which 478 were ESBL, thus amounting to a prevalence of 50.80%. The genetic profiles of the bla CTX-M resistance genes exhibited the emergence of the CTX-M28 variant (CTX-M1 group) and variants of the M2 and M9 groups. There was also a notable increase, from 35 to 64%, in the ESBL with a bla SHV gene.

**Conclusion:**

this study documents a 13 fold increase in the prevalence of ESBL derived from clinical isolates of the bacteriology laboratory of the Institute Pasteur in Bangui, by comparing its data with that of the publication by Frank et al. 2006. Together with this increase a significant diversification of the circulating CTX-M resistance genes was noticed.

## Introduction

The development and use of antibiotics over the past 70 years has allowed for a major reduction in the mortality and morbidity associated with bacterial infectious diseases throughout the world. However, although antibiotic drugs have saved the lives of millions of patients, their extensive and sometimes uncontrolled use has promoted the emergence of bacteria resistant to an increasing number of antibiotics. In developing countries (DC), poverty, malnutrition, poor sanitation, and a lack of organized and efficient health care systems have promoted the occurrence and the spread of resistance to antibiotics, thereby affecting the most frequently isolated pathogenic agents in hospitals and in the community setting. Government crises, as well as civil wars and the displacement of populations, which are frequent in these parts of the world, are factors that amplify the occurrence of antibiotic resistance [1,2]. The production of extended-spectrum beta-lactamases (ESBL) is the most common multidrug resistance mechanism in enterobacteria [3]. These enzymes, which hydrolyze extended-spectrum penicillins and cephalosporins, initially came from narrow-spectrum plasmid penicillinases and were mainly found in hospital strains of *Klebsiella pneumoniae* [4]. As of 1995, a plethora of “new” ESBL of the CTX-M type have emerged, and they have completely changed the epidemiological situation at the global level. Indeed, they have now spread to all species of enterobacteria, in hospitals as well as in the community, as a result of an “epidemic of plasmids” and/or other mobile genetic elements, combined with clonal expansion [5]. Moreover, these plasmids carrying ESBL genes also harbor other resistance genes that confer the majority of ESBL enterobacteria resistance to other families of antibiotics, particularly cotrimoxazole, fluoroquinolones, and aminosides [6].

There is a paucity of data regarding the spread of ESBL in community and hospital environments in Africa. In Chad, in 2017, a study involving 100 healthy community volunteers and 100 hospitalized patients found that 44.5% of the fecal enterobacteria were ESBL (51% in the hospitalized patients and 38% in the healthy volunteers) [7]. A similar study has been undertaken in Burkina Faso (2014), in which 32% of the isolated bacteria were carriers (22% in the volunteers and 42% in the hospitalized patients) [8]. In Madagascar (2011), the rate was 31% at admission among patients, 75% of whom had not previously been hospitalized, thus suggesting acquisition of resistance in the community setting [9]. In Central Africa, the data published by Frank *et al*. revealed 3.78% ESBL-positivity among the enterobacteria in the clinical isolates of the bacteriology laboratory of the Pasteur Institute in Bangui (PIB), between 2003 and 2005 [10]. The main objective of the present study was to update this data by analysis of the enterobacterial isolates of 2018 and 2019, obtained at the PIB by recruitment of patients and by bacteriological techniques that were very comparable for the two periods. The publication by Frank *et al*. in 2006 also described the diversity of the *bla CTX-M, bla TEM*, and *bla SHV* beta-lactamase genes; these characteristics were also compared with a panel of ESBL strains from 2017 and 2018 investigated in the context of a nosocomial epidemic in a maternity ward in Bangui (unpublished data).

## Methods

**Type of study, population, and period:** this was a retrospective study from May 2018 to April 2019 involving the patients sent to the clinical laboratory at the Pasteur Institute in Bangui for bacterial testing of biological samples (urine, stools, vaginal and cervical swabs, sperm, puncture fluids, blood cultures, and samples from discharges and lesions). For the genotyping, 38 strains of *Klebsiella pneumoniae* were studied: sixteen (16) derived from blood cultures of neonates in the context of a nosocomial epidemic (2017) and 22 derived from rectal samples from pregnant women (2018).

**Criteria for inclusion:** all patient files comprising bacteriological testing with the isolation of at least one type of enterobacterium were included.

**Methodology:** culturing and isolation of the enterobacteria were carried mainly on bromo cresol-Purple (BCP), Hektoen, or Chocolate agar (Bio-Rad, Marnes la Coquette, France). The phenotypic identifications were carried out using the biomerieux API 20E panel and API Web Standalone 1.3.2. Software. The sensitivities to antibiotics were determined using the Mueller-Hinton agar diffusion method (Bio-Rad antibiotic discs, Marnes la coquette, France) and interpreted according to the 2018 and 2019 guidelines of the Antibiogram Committee of the French Society of Microbiology (CA-SFM EUCAST). The enterobacteria producing extended-spectrum beta-lactamases (ESBL) were selected based on the following criteria: (i) susceptibility to cefoxitin and reduced sensitivity to cefotaxime (5 μg), ceftazidime (10 μg), ceftriaxone (30 μg), and cefepime (30 μg) (diameter of the area of inhibition < 21mm) and (ii) enhanced susceptibility in the presence of the association of amoxicillin/clavulanic acid and a third-generation cephalosporin, determined by the double-disc synergy test. The molecular analyses were carried out using pure bacterial cultures of 24 hours. The deoxyribonucleic acid (DNA) extraction method was based on heat shock: heating to 100°C for 5 minutes, followed by centrifugation at 13,000 rpm for 10 minutes. The supernatant was collected and aliquoted ready for use, or stored at -20°C. Simplex end-point polymerase chain reaction (PCR) was performed to characterize the *bla* CTX-M, *bla* TEM, and *bla* SHV resistance genes. The reaction volume was 50 μl, with the following concentrations: 10x PCR + Magnesium chloride (MgCl_2_) Taq PCR Core buffer, 10mm dNTPs (deoxyribonucleotide triphosphate) mix, 20 pmol (picomoles per litre)/μl primers, 5 IU/μl Taq DNA polymerase, and 2 μl of the extracted DNA. Amplification of the genes was carried out with an Applied Biosystems Gene Amp 9700 thermocycler according to the programs listed in Table 1. The specific primer pairs that were used are presented in Table 2. The amplification products were analyzed by electrophoresis on 1.5% agarose gels to which 1X GelRed® had been added to reveal the amplicons. Migration of the products was carried out at 120 volts for 1 hour in 1X TAE buffer. An utralviolet (UV) lightbox (U: GENIUS3, Syngene) was used to visualize and image the gel. The PCR products were sequenced by the Sanger technique subcontracted to GATC-Eurofins (Köln, Germany). The DNA sequences were analyzed using BLAST software. The ethics and scientific committee of the Faculty of Sciences-University of Bangui issued a favorable opinion for the publication of the results of this retrospective study, considering that the ethical principles of research on human beings have been respected. Comparison of the proportion of ESBL phenotypes between 2003-2005 and 2018-2019 was carried out with the Chi-2 test or Fisher´s exact test using MedCalc software (MedCalc Statistical Software version 19.1.5). Differences between the compared proportions were considered statistically significant when the p-value was less than 0.05.

**Table 1:** real-time PCR programs for *bla* CTX, TEM and SHV genes

	Initial denaturation			Denaturation			Hybridization			Elongation			Extension		
Genes:	T° (C)	Time (min)	# of Cycles	T° (C)	Time (min)	# of Cycles	T° (C)	Time (min)	# of Cycles	T° (C)	Time (min)	# of Cycles	T° (C)	Time (min)	# of Cycles
***bla* CTX-M1, M2 and M9**	94	5	1	94	1	30	60	1	30	72	1	30	72	1	30
***bla* CTX-M8, *bla* SHV**	94	5	1	94	1	30	58	1	30	72	1	30	72	7	30
***bla* TEM**	94	5	1	94	1	30	50	1	30	72	1	30	72	7	30

**Table 2:** primers used to detect β-lactam resistance genes

Primers	Sequences (5´-----> 3´)	Hybridization T°	Product size (bp)
Ctx-M1 (+)	GGTTAAAAAATCACTGCGTC	60 °C	863
Ctx-M1 (-)	TTGGTGACGATTTTAGCCGC		
Ctx-M2 (+)	ATGATGACTCAGAGCATTCG	60 °C	865
Ctx-M2 (-)	TGGGTTACGATTTTCGCCGC		
Ctx-M8 (+)	GCGGCGCTGGAGAAAAGCAG	58 °C	608
Ctx-M8 (-)	GCTGCCGGTTTTATCCCGA		
Ctx-M9 (+)	ATGGTGACAAAGAGAGTGCA	60 °C	869
Ctx-M9 (-)	CCCTTCGGCGATGATTCTC		
TEM-OT3	TTACCAATGCTTAATCCACA	50 °C	863
TEM-OT4	TAAAATTCTTGAAGATGCCG		
SHV-OS5	GATTTGCTGATTTCGCTCGG	58 °C	857
SHV-OS6	TTATCTCCCTGTTAGCCACC		

## Results

**Phenotypic analyses:** between 01/05/2018 and 30/04/2019, the clinical lab of the Pasteur Institute in Bangui recorded 7,149 biological analysis files comprising at least one bacteriological examination (Table 3). In 927 of these files, cultivation led to the isolation and identification of at least one enterobacterial strain recognized as potentially pathogenic, and for which an antibiogram was hence performed. Since some of the samples led to the isolation of two or more enterobacteria, 941 enterobacteria were ultimately included in this study. Of these 941 strains, 478 (50.79%) had the ESBL phenotype, of which 166 (34.73%) were from urine cultures, 154 (32.22%) were from coprocultures, 32 (6.69%) were from blood cultures, 24 (5.02%) were from genital samples, and 102 (21.34%) from other sites (e.g pus, lesions, puncture fluids). The bacterial species involved were *Escherichia coli* (263 or 55.02%), *Klebsiella pneumoniae* (119 or 24.90%), *Klebsiella oxytoca* (34 or 7.11%), *Enterobacter cloacae* (29 or 6.07%), and other species of enterobacteria (33 or 6.90%).

**Table 3:** the prevalence of the enterobacteria with an ESBL phenotype among the clinical isolates of the LMA of the Pasteur Institute of Bangui for 2003-2005 and 2018-2019

Study period		2003-2005		2018-2019		P
Number of enterobacteria studied		450	100.00%	941	100.00%	-
Number of extended-spectrum beta-lactamases (phenotype)		17	3.78%	478	50.80%	< 0.0001
Of which:	*E. coli*	10	58.82%	263	55.02%	NS (> 0.05)
	*K. pneumoniae*	4	23.53%	119	24.90%	NS (> 0.05)
	Other species	3	17.65%	81	16.95%	NS (> 0.05)

**Genotypic analyses:** the 38 strains tested by PCR all had at least one *bla* CTX-M gene (Table 4). The distribution of the groups and the associations were as follows: 29 strains (76%) had the CTX-M1 group only, 1 strain (3%) had the CTX-M2 group only, 7 strains (18%) had both CTX-M1 and CTX-M2, and 1 strain (3%) had both CTX-M2 and CTX-M9. Ten strains with the CTX-M1 group were sequenced: the variants found were CTX-M15 (4 strains) and CTX-M28 (6 strains). Polymerase chain reaction was used to detect the *bla* CTX-TEM and *bla* CTX-SHV genes in 22 strains, and these genes were found to be present in 22 (100%) and 14 (64%), respectively.

**Table 4:** genetic profiles of the enterobacteria with an ESBL phenotype among the clinical isolates of the clinical lab of the Pasteur Institute of Bangui for 2003-2005 and 2018-2019

Gene	Group (PCR)	Variant (sequence)	2003-2005		2017-2018	
**Number**			17		38 (22 for TEM - SHV)	
***bla* CTX-M**	CTX-M1	M-15	11	65%	4	11%
		M-3	1	6%	0	0%
		M-28	0	0%	6	16%
		not sequenced	0	0%	26	68%
		**Total M1 group:**	**12**	**71%**	**36**	**95%**
	CTX-M2	not sequenced	0	0%	9	24%
	CTX-M8	not sequenced	0	0%	0	0%
	CTX-M9	not sequenced	0	0%	1	3%
	**Total CTX-M:**		**12**	**71%**	**46**	121%*
***bla* TEM**		TEM 1	17	100%	0	0%
		not sequenced	0	0%	22	100%
	**Total TEM:**		**17**	**100%**	**22**	**100%**
***bla* SHV**		SHV 12	4	24%	0	0%
		SHV 2a	2	12%	0	0%
		not sequenced	0	0%	14	64%
	**Total SHV:**		**6**	**35%**	**14**	**64%**

## Discussion

The main objective of this study was to evaluate the change in the prevalence and the genetic diversity of ESBL circulating in the Central African Republic (CAR) in 2003-2005 (initial publication from our laboratory [10]) and 2018-2019. As the recruitment of bacterial strains and the techniques employed in these two periods were very similar, the observed differences are consequently not a reflection of procedural differences. Table 3 and Table 4 present a comparison of the phenotypic and the genetic data of the two studies. As the prevalence of ESBL went from 3.78% to 50.80%, it increased 13.4-fold between 2003-2005 and 2018-2019, thus amounting to a highly significant increase (p < 10-4). On the other hand, there was little change in the ESBL distribution in terms of the bacterial species, thus indicating that the recruitment was comparable for these two periods, with *Escherichia coli* and *Klebsiella pneumoniae* being the most frequently encountered species. The prevalence of ESBL in the present study (50.80%) is similar to what has been reported in several African countries, such as Ghana 49.4% [11], Gabon 50% [12], Burkina Faso 58% [13], Cameroon 55.3% [14], and Chad 47.7% [15]. These bacteria have hence become widespread on the African continent. The high level of resistance can be explained by the common use of beta-lactam antibiotics, which are frequently provided as first-intention treatment of infections by enterobacteria. Other factors also come into play, such as self-medication; prescriptions that are not based on the bacteriology due to the absence of available means for a biological diagnosis; poor hygiene, both at the community and hospital level; and uncontrolled circulation of counterfeit medications [16,17]. Although the genotyping was only carried out on a small number of strains, the genotypic profiles of the ESBL enzymes encountered in these two periods suggest substantial diversification of these enzymes. In 2003-2005, only the CTX-M1 group, with a predominance of the M-15 variant, was noted among the *bla* CTX-M genes; in 2018-2019, the CTX-M2 and M9 groups emerged, and in CTX-M1 group, along with the high-prevalence M-15 variant, the M-28 variant was identified. Since the start of the 2000s, the M-15 variant had been widely described in Africa in enterobacteria that produce ESBL, particularly in South Africa [18], in Kenya [19], in Senegal [20] in Cameroon [21] in Tanzania [22], and in Nigeria [23]. On the other hand, the M-28 variant, which is genetically close to M-15, has been reported less in the literature. Since its discovery in France in 2003, it has been reported in China, India, Korea, and Brazil [24]. In Africa, this variant has only been reported for a single case in Tunisia in 2009 [25]. In 2016, in the United Arab Emirates, it was found to be dominant and it appears to have replaced the M-15 variant [26], which is similar to the situation reported since 2009 in Korea [27]. For the profiles obtained at Bangui in 2018-2019, the presence of both the M-15 and the M-28 variant could be indicative that a replacement (shift) is taking place, which would warrant being closely monitored. To corroborate this current diversity of the ESBL enzymes identified in 2017-2018, it should be pointed out that, unlike in 2003-2005, strains that concurrently had two *bla* CTX-M genes were shown to occur in the later period, and that the frequency of SHV genes had increased from 35 to 64% in the intervening years.

## Conclusion

In a 12-year period, the observed prevalence of ESBL among the enterobacteria of clinical isolates increased 13-fold in the CAR, reaching a prevalence rate of 50.8% in 2018, which is comparable to the rates observed elsewhere on the African continent. This increase was accompanied by a substantial degree of diversification of the observed *bla* genes, with the emergence of variants of the CTX-M2 and M9 groups, as well as the CTX-M-28 variant (CTX-M1 group), possibly replacing the high-prevalence M-15 variant. This change needs to be closely monitored, as it could potentially occur quite fast. These findings reflect a negative impact on the mortality and the morbidity associated with bacterial infections in the CAR, as they indicate a therapeutic stalemate and the probable emergence of other resistance phenotypes, such as strains that produced carbapenemases, which should also be the subjected to specific monitoring. Controlling this situation involves targeted public health actions, aimed particularly at (i) education of the medical body (good practices for the prescription of antibiotics), (ii) control of medications (limitation of free circulation and self-medication, combating counterfeits), (iii) improvement of sanitary conditions in general (oral-fecal hygiene, access to care) and in hospitals (combating nosocomial infections), (iv) access to bacteriological diagnoses, and (v) control of the use of antibiotics for veterinary purposes. Among the indicators of performance for these measures, it has been proposed to set up an antibiotic resistance monitoring center, based initially on the data from the clinical laboratory of the Pasteur Institute in Bangui, and then on the input from other bacteriological laboratories in the country when they become operational. Its objective would be to follow the progression of multiresistant bacteria in real-time and to allow early detection of the emergence, for example, of strains that produce carbapenemases.

### What is known about this topic

- The emergence of ESBLs in CAR has been reported since the early 2000s and more generally in the Central and Western African region;
- The genetic variant most frequently associated with these ESBLs was historically the CTX-M-15 variant of the CTX-M1 group.

### What this study adds

- In 14 years (from 2004 to 2018), the prevalence of ESBL among clinical isolates of enterobacteria increased 13-fold in CAR, confirming the massive spread of these multidrug-resistant bacteria;
- This increase is accompanied by a significant diversification of the genetic variants involved, with a possible replacement in progress of variant CTX-M15 by CTX-M28.
